# The peptide agonist-binding site of the glucagon-like peptide-1 (GLP-1) receptor based on site-directed mutagenesis and knowledge-based modelling

**DOI:** 10.1042/BSR20150253

**Published:** 2016-01-15

**Authors:** Rachel L. Dods, Dan Donnelly

**Affiliations:** * School of Biomedical Sciences, Faculty of Biological Sciences, University of Leeds, Leeds LS2 9JT, U.K.

**Keywords:** diabetes, GLP-1, GPCR, hormone, molecular model, receptor

## Abstract

Glucagon-like peptide-1 (7–36)amide (GLP-1) plays a central role in regulating blood sugar levels and its receptor, GLP-1R, is a target for anti-diabetic agents such as the peptide agonist drugs exenatide and liraglutide. In order to understand the molecular nature of the peptide–receptor interaction, we used site-directed mutagenesis and pharmacological profiling to highlight nine sites as being important for peptide agonist binding and/or activation. Using a knowledge-based approach, we constructed a 3D model of agonist-bound GLP-1R, basing the conformation of the N-terminal region on that of the receptor-bound NMR structure of the related peptide pituitary adenylate cyclase-activating protein (PACAP21). The relative position of the extracellular to the transmembrane (TM) domain, as well as the molecular details of the agonist-binding site itself, were found to be different from the model that was published alongside the crystal structure of the TM domain of the glucagon receptor, but were nevertheless more compatible with published mutagenesis data. Furthermore, the NMR-determined structure of a high-potency cyclic conformationally-constrained 11-residue analogue of GLP-1 was also docked into the receptor-binding site. Despite having a different main chain conformation to that seen in the PACAP21 structure, four conserved residues (equivalent to His-7, Glu-9, Ser-14 and Asp-15 in GLP-1) could be structurally aligned and made similar interactions with the receptor as their equivalents in the GLP-1-docked model, suggesting the basis of a pharmacophore for GLP-1R peptide agonists. In this way, the model not only explains current mutagenesis and molecular pharmacological data but also provides a basis for further experimental design.

## Abstract (precis)

Mutagenesis and molecular pharmacological analysis of the glucagon-like peptide-1 (GLP-1) receptor highlighted several residues involved in peptide agonist recognition. Coupled with a new molecular model of the full-length agonist-docked receptor, the binding site and a pharmacophore for agonist peptides are described.

## INTRODUCTION

**Figure 1:**
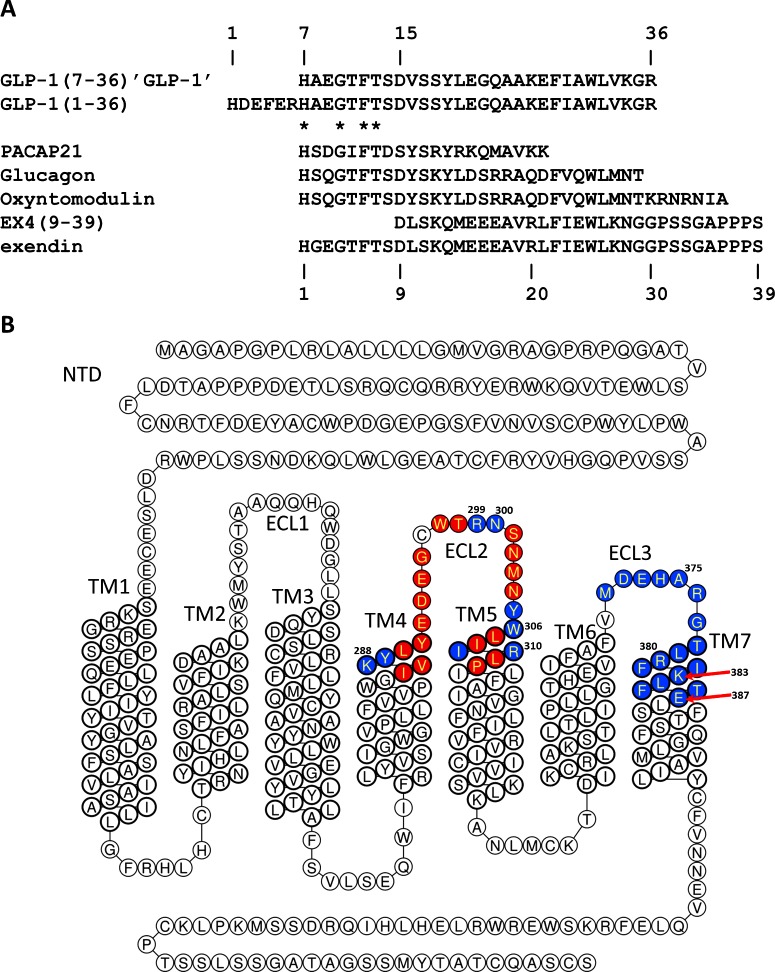
Sequence and numbering of ligands and receptor residues discussed in this paper (**A**) The aligned amino acid sequences of the peptides discussed in the present paper. Note that GLP-1 starts with residue His-7*, whereas the remaining peptides begin at residue 1. (**B**) A schematic topological representation (generated using GPCRDB Tools, http://tools.gpcr.org/) of GLP-1R, annotated to show the regions mutated in the present study (blue: single mutations, data in main body of paper; red: remaining sites from the initial screen of double mutations, data in Supplementary Material). Residue numbers of the most interesting sites are highlighted.

Glucagon-like peptide-1 (GLP-1) is an ‘incretin’ hormone of 30 residues (Figure 1A) which is released from intestinal L-cells in response to feeding, whereby it acts at pancreatic β-cells to potentiate insulin secretion in a glucose-dependent manner [1–3]. The hormone's central role in post-prandial insulin release, alongside its other effects such as the inhibition of gastric emptying [4], the inhibition of glucagon secretion [5] and the reduction in food intake [6], have resulted in the receptor for GLP-1 (GLP-1R) becoming a major target for the potential treatment of diabetes. Indeed, there are two peptides (exenatide and liraglutide) which are already licensed for use in therapy [7,8].

GLP-1R is a typical ‘Family B’ G protein-coupled receptor (GPCR), a family characterized by an extracellular N-terminal domain (NTD) of 100–150 residues and a TM domain (7TM domain) consisting of seven TM α-helices (TM1–TM7), the six connecting loops and a C-terminal tail [9] (Figure 1B). The proposed model for agonist-induced activation of Family B GPCRs, including GLP-1R, is a two-step mechanism in which the C-terminal half of the peptide hormone's α-helix binds to the NTD, whereas a second interaction between the N-terminal residues of the ligand and the 7TM of the receptor leads to receptor activation [10]. Several X-ray structures of the isolated NTDs of Family B GPCRs have shown that they share a common fold stabilized by three conserved disulphide bonds consisting of an N-terminal α-helix and a short consensus repeat [11–14]. More recently, the first crystal structures of the 7TM domain of two Family B GCPRs, solved in the absence of both the NTD and the peptide ligand, have recently been described [15,16].

The NTD of GLP-1R binds to the helical region of GLP-1 (residues 24*–33*; residues in the ligand will be distinguished from those in the receptor throughout the paper by the addition on an asterisk *) via a shallow groove on the surface of the domain and the details of this interaction are well understood via X-ray crystallography [13,14, reviewed in 17]. However, despite the recent publication of the crystal structure of the TM domain of the closely related glucagon receptor (GlucR; [16]), the details of the interaction between GLP-1’s N-terminal region and the receptor remain largely unknown. There are however some data which inform us of the likely conformation of the N-terminal region of the active GLP-1 peptide. Firstly, Inooka et al*.* [18] published the receptor-bound structure of the related peptide pituitary adenylate cyclase-activating protein (1–21) amide (PACAP21), solved by proton NMR (2D TRNOE; pdb code 1GEA), which showed that residues 3*–7* formed a β-coil structure preceded by an extended N-terminal tail. The N-terminal region of GLP-1 is closely related to that of PACAP (Figure 1A) and may therefore fold in a similar manner. Secondly, Hoang et al. [19] have recently published the NMR structures of several 11-residue analogues of GLP-1 containing cyclic constraints. One such peptide, containing a disulphide link between homocysteine residues at positions 2* and 5* (equivalent to residues Ala-8* and Thr-11* in GLP-1), maintained sub-nanomolar potency in cAMP assays and was shown by NMR to have a type II β-turn type (pdb code 2N0I), which was also observed in the non-constrained parent compound.

The aim of this work was to determine a detailed working molecular model for agonist-docked GLP-1R that accounts for our current knowledge and that can also act as a basis for the design of new ligands and further experiments. Following a review of the published literature relating to the site-directed mutagenesis of GLP-1R (Supplementary Figure S2; Supplementary Table S1), we designed an Ala-scan mutagenesis approach targeted at a 17-residue region of the receptor based around the 3rd extracellular loop (ECL3) and the neighbouring region of TM7 (Figure 1b). Mutated receptors were expressed in human embryonic kidney (HEK)293 cells and analysed using both radioligand-binding analysis to assess affinity, and cAMP accumulation assays to assess efficacy. Further sites in ECL2 and TM5 were targeted in a similar manner (Figure 1). A molecular model of the full-length peptide-bound GLP-1R was generated using a knowledge-based approach by combining three components: the crystal structure of the NTD bound to GLP-1; a homology model of the 7TM domain of GLP-1R based upon the closely related glucagon receptor crystal structure and a homology model of the N-terminal region of GLP-1 based upon the receptor-bound structure of the related peptide PACAP21 solved via NMR [14,16,18]. The mutagenesis data published here, alongside that from the literature, were used to inform the docking of the ligand and to suggest the key interaction sites required for agonist binding and activation. To validate the model, the structure of a cyclic constrained 11-residue GLP-1 analogue ([19]; pdb code 2N0I), which has a different conformation to that determined for receptor-bound structure of the related peptide PACAP21 ([18]; pdb code 1GEA), was docked into the GLP-1R model so that a pharmacophore for peptide agonists could be determined.

## MATERIALS AND METHODS

### Constructs

The pcDNA5-FRT vector (Invitrogen) containing the full-length human GLP-1R [10], was used to express the wild-type receptor. The mutated cDNA used to express the mutant receptors were generated using QuikChange site-directed mutagenesis (Stratagene), and confirmed by DNA sequencing. These constructs were used to express the wild-type and mutant GLP-1 receptors in Flp-In HEK293 cells (Invitrogen).

### Cell culture

The Flp-In HEK293 cells were cultured in Dulbecco's modified Eagle's medium (Sigma) supplemented with 10% foetal calf serum (Lonza Wokingham Ltd.), 2 mM L-glutamine, 100 unit/ml penicillin and 100 μg/ml streptomycin (Invitrogen). Cells were transfected with the pcDNA5.FRT vector and pOG44 using Lipofectamine® 2000 transfection reagent (Invitrogen) and stable isogenic clones were selected by the addition of the antibiotic hygromycin (Sigma) at a concentration of 100 μg/ml.

### Peptides

GLP-1(7–36)amide (GLP-1) and exendin-4(9–39)amide [EX4(9–39)] were purchased from Bachem (Saffron Walden). ^125^I-Bolton-Hunter labelled EX4(9–39) was purchased from PerkinElmer. The radioligand ^125^I-GLP-1 was the kind gift of Novo Nordisk (Copenhagen).

### Radioligand binding

Flp-In HEK293 cells, cultured to confluence on five 160-cm^2^ Petri dishes (pre-coated with poly-D-lysine), were washed with PBS, followed by the addition of 15 ml of ice-cold sterile double distilled water to induce cell lysis. Following 5 min incubation on ice, the ruptured cells were thoroughly washed with ice-cold PBS before being scraped from the plates and pelleted by centrifugation in a bench-top centrifuge (13,000 *g* for 30 min). The crude membrane pellet was resuspended in 1 ml membrane-binding solution (MBS; 50 mM HEPES pH 7.4, 2.5 mM CaCl_2_, 5 mM MgCl_2_, 0.2% BSA) and forced through a 23G needle. Aliquots (0.1 ml) were snap-frozen in liquid nitrogen and stored at −70°C. When required for assay, membranes were slowly thawed on ice before diluting to a concentration that gave total radioligand binding of <10% total counts added. In a reaction volume of 200 μl, 75 pM (∼60,000 cpm) of radioligand [^125^I-GLP-1 or ^125^I-exendin(9–39)], various concentrations of an unlabelled competitor ligand and HEK293 membranes expressing the receptor of interest were combined, all diluted in MBS. Assays were carried out for 1 h in MultiScreen 96-well Filtration Plates (Glass fibre filters, 0.65 μm pore size, Millipore) pre-soaked in 1% non-fat milk/PBS. After the incubation, membrane-associated radioligand was harvested by transferring the assay mixture to the filtration plate housed in a vacuum manifold. The wells of the filtration plate were washed three times with washing buffer (0.2 ml PBS, 0.1% BSA) before harvesting the filter discs. Filter-bound radioactivity was measured in a γ-counter (RiaStar 5405 counter; PerkinElmer Life and Analytical Sciences). Total radioligand bound was <10% and non-specific binding was ∼1% of total counts added.

### cAMP assays

The LANCE cAMP kit (PerkinElmer Life and Analytical Sciences) was used alongside the manufacturer's instructions with some minor adaptations as described. Flp-In HEK293 cells expressing the receptor of interest were washed and resuspended in stimulation buffer: HBSS, 5mM HEPES, 0.1% BSA, 500 μM IBMX (all from Sigma), pH 7.4, at a concentration of 1×10^6^ cells/ml. Cell numbers were set at 2500 cells/well based upon previous experiments (data not shown), in order that raw fluorescence data fell within the linear range determined by a standard cAMP concentration curve. The Alexa Fluor® 647 labelled antibody was added to the cell suspension at a final concentration of 0.005% (v/v). Ligand concentrations were used in the range of 100 μM to 1 pM made up in the vehicle (stimulation buffer). The ligand was added as 6 μl/well of each concentration (in triplicate) to a white 384 well low volume OptiPlate (Greiner). To this was added 6 μl of the prepared cell suspension and the contents of the plate were mixed, sealed and left for 10 min at 37°C. The detection mixture was prepared in a separate tube by diluting the Eu-W8044 labelled streptavidin 2250-fold in the detection buffer supplied with the kit. The Biotin-cAMP was then added such that is was diluted 750-fold. This mixture was incubated for at least 30 min at room temperature to allow complex formation to occur. Once the 10 min agonist stimulation time was complete, 12 μl of the detection mixture was added to each well and incubated at room temperature for 1 h. The acceptor fluorescence signal was then read at 665 nm using Victor TM X4 2030 multi-label plate reader (PerkinElmer).

### Data analysis

Binding and cAMP assays were carried out with triplicate values at each ligand concentration and with each assay being repeated at least three times. The resultant data were analysed using Prism 6 (GraphPad Software Inc.). IC_50_ values were calculated from the competition binding data using non-linear regression with a three-parameter logistic equation, and then converted to dissociation constant *K*_i_, using the Cheng and Prusoff equation, by first calculating the *K*_i_ for exendin(9–39) from homologous competition assays and then using this value to calculate *K*_i_ for GLP-1 from heterologous competition assays [20]. Expression levels (*B*_max_) were estimated from the homologous competition assays using *B*_max_=*B*_0_ × IC_50_/[*L*], where [*L*] is the concentration of free radioligand and *B*_0_ is the specific binding in the absence of unlabelled ligand. *B*_max_ values were expressed as fmol of receptor/mg of membrane protein where the latter was calculated using a bicinchoninic acid protein assay using BSA to create a standard curve. The cAMP assay data were analysed using both a logistical and operational model in order to determine potency (EC_50_) and efficacy (*τ*) (see [21] for equations). When using the operational model to determine efficacy, the *K*_i_ values from the corresponding binding analyses were used as a constraint during the non-linear regression. The estimated *τ* values were then normalized to cell surface expression using the *B*_max_ values determined from the binding analysis to give *τ*_c_ (errors from both the *τ* and *B*_max_ estimations were pooled). Efficacies for mutants were compared with wild-type GLP-1R by comparing log *τ*_c_ values using an unpaired two-tailed Student's *t* test (unequal variance), with *P*<0.005 being used as a threshold for a significant reduction in efficacy.

### Molecular modelling

All molecular modelling manipulations (Supplementary Figure S1) were carried out using the tools embedded within PyMol (The PyMOL Molecular Graphics System, Version 1.7.2.3 Schrödinger, LLC) unless otherwise stated. (1) Model 1 from the ensemble of NMR structures for the receptor-bound structure of C-terminally truncated PACAP ([18]; pdb code 1GEA) was (*in silico*) mutated to the GLP-1 sequence and (2) this was then structurally aligned with the GLP-1 molecule within the NTD structure of GLP-1R ([14]; pdb code 3IOL) by superimposing residues Val-16*–Gly-22*. (3) The structure of the antagonist-bound structure of the NTD structure of GLP-1R ([13]; pdb code 3C5T) was structurally aligned to the product of stage 2. (4) The following residues and moieties were then removed from the product of stage 3: GLP-1 10*–21* from 3IOL; GLP-1 22*–29* from the mutated 1GEA; all of 3C5T except for residues Ser-129–Arg-131; all waters and other non-protein atoms. The remaining atoms were saved to a single pdb file which now represented a model of the NTD of GLP-1R with GLP-1 bound, as observed in 3IOL, but with three extra residues fused on to the C-terminus (Ser-129–Arg-131 from 3C5T) and an N-terminally extended version of the GLP-1 ligand having the conformation of His-7*–Glu-21* based upon the receptor-bound PACAP21 structure. (5) The structure of the 7TM domain of the glucagon receptor ([16]; pdb code 4L6R) was used as a template to model the equivalent region of GLP-1R using the homology modelling server SWISS-MODEL ([22]; http://swissmodel.expasy.org/). ECL1 was not modelled as it was absent from the template structure. (6) The surface of the product of stage 5 was displayed in PyMol using the ‘Cavities and Pockets Only’ setting, in order to aid the manual docking of the product of stage 4 as a rigid body by inserting the N-terminal region of the peptide ligand into the highlighted cavity of the 7TM domain. During this docking process, the mutagenesis data from the literature were carefully considered (Supplementary Figure S2; Supplementary Table S1) and some local side-chain rotamers were modified and subjected to local optimization using the ‘Sculpting’ tool in PyMol. Once the docking had been finalized, the atoms of both components were saved as a single pdb file and then subjected to optimization using the KoBa^MIN^ server ([23]; http:://csb.stanford.edu/kobamin) to yield the final model.

## RESULTS

### ECL3/TM7

**Figure 2:**
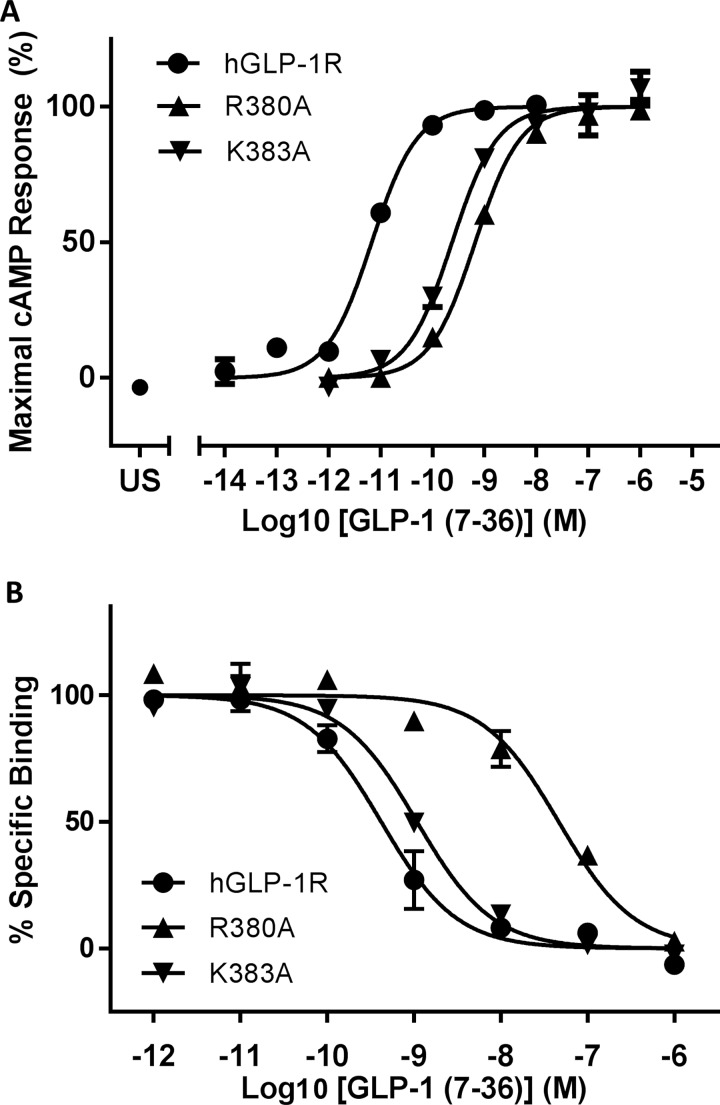
Pharmacological analysis of two TM7 single mutant receptors compared with wild-type GLP-1R: Arg-380^7.34^–Ala, Lys-383^7.37^–Ala (**A**) Concentration–response curves for GLP-1-induced cAMP accumulation in Flp-In HEK293 cells expressing either hGLP-1R or the mutated receptors. US, unstimulated. (**B**) Radioligand competition binding curves, using 75 pM ^125^I-EX4(9–39) and unlabelled GLP-1 as competitor, for membranes derived from Flp-In HEK293 cells expressing either wild-type GLP-1R or mutated receptors. Replicates are in triplicate with errors representing S.E.M.

A total of 17 single site-directed mutations of residues spanning Met-371^ECL3^ to Glu-387^7.41^ (ECL3/TM7; superscripts refer to Wootten numbering [21] for TM residues, or else identify the region in which the residue is located; Figure 1B) were generated using QuikChange mutagenesis. Stable Flp-In HEK293 cell lines were selected for each mutant receptor and both LANCE cAMP assays and radioligand competition assays were carried out in order to estimate their pharmacological properties (Table 1). GLP-1 acting at Arg-380^7.34^–Ala had a 240-fold reduced potency, with a 129-fold reduced affinity; whereas at Lys-383^7.37^–Ala GLP-1 had 51-fold reduced potency but affinity that was not significantly different from wild-type GLP-1R (Figure 2). However, although the operational model demonstrated that Lys-383^7.37^–Ala had significantly reduced efficacy (Δlog *τ*_c_=1.18), this was not the case for Arg-380^7.34^–Ala where the reduced potency could be accounted for by the reduced affinity. GLP-1 acting at Glu-387^7.41^–Ala had reduced efficacy (Δlog *τ*_c_=0.52), whereas Ala-375–Gly^ECL3^ displayed 10-fold reduced affinity. Asp-372–Ala^ECL3^ and Leu-284^7.38^–Ala displayed reduced potency but did not have significantly reduced affinity or efficacy for GLP-1.

**Table 1:** Pharmacological properties of site-directed mutations of GLP-1R with asterisk (*) representing data discussed in the text Numbers in brackets refer to fold-change relative to wild-type (WT) GLP-1R if statistically different from WT GLP-1R. ^†^*P*<0.005, representing a significant reduction in *p*EC_50,_ *p*IC_50_ or log *τ*_c_ using GLP-1

Mutant	GLP-1 *p*EC_50_	GLP-1 log *τ*_c_	GLP-1 *p*IC_50_	EX4(9–39) *p*IC_50_	*B*_max_ (pmol/mg)
WT GLP-1R*	11.38±0.15*	1.97±0.14*	9.45±0.09*	8.35±0.31*	21.8±1.5*
K288A^4.46*^	7.66±0.10 (5248)^*†^	0.58±0.02^*†^	8.09±0.08 (23)^*†^	8.20±0.11	18.1±0.1
Y289A^4.65^	10.38±0.17	1.89±0.14	9.33±0.04	8.48±0.12	4.3±0.2
R299A^ECL2*^	10.50±0.15	1.37±0.08^*†^	9.87±0.03	8.59±0.03	8.8±0.2
N300A^ECL2*^	9.40±0.06 (95)^*†^	2.28±0.06	7.90±0.10 (36)^*†^	8.53±0.16	3.9±0.3
Y305A^5.35^	10.70±0.05 (5)^*†^	2.16±0.06	9.26±0.06	8.29±0.11	4.9±0.3
W306A^5.36 *^	9.10±0.28 (191)^*†^	2.09±0.19	7.41±0.05 (110)^*†^	8.61±0.09	9.0±0.4
I309A^5.39^	10.20±0.41	1.53±0.29	8.93±0.06	8.31±0.30	16.4±0.8
R310A^5.40*^	8.31±0.03 (1175)^*†^	1.22±0.04^*†^	8.91±0.02	8.37±0.33	4.1±0.7
M371A^ECL3^	10.88±0.14	2.49±0.17	8.65±0.06	8.26±0.12	11.8±0.1
D372A^ECL3^	9.65±0.11 (54)^*†^	1.84±0.10	8.85±0.15	8.25±0.06	2.9±0.0
E373A^ECL3^	11.16±0.03	2.26±0.02	9.46±0.05	8.39±0.23	6.0±0.0
H374A^ECL3^	11.43±0.11	2.53±0.13	9.57±0.05	8.32±0.18	4.8±0.4
A375G^ECL3*^	10.52±0.16	1.78±0.21	8.46±0.05 (10)^*†^	7.67±0.32	40.5±1.6
R376A^ECL3^	11.32±0.06	1.94±0.12	9.23±0.02	8.13±0.14	29.9±2.2
G377A^ECL3^	11.01±0.11	2.67±0.16	9.03±0.05	8.32±0.32	4.1±0.4
T378A^7.32^	11.19±0.02	2.65±0.02	9.50±0.13	8.31±0.26	2.7±0.2
L379A^7.33^	11.08±0.07	2.49±0.11	9.08±0.03	8.17±0.07	6.8±1.1
R380A^7.34*^	9.00±0.05 (240)^*†^	1.79±0.06	7.34±0.01 (129)^*†^	7.92±0.07	14.8±0.1
F381A^7.35^	11.18±0.10	2.72±0.11	9.23±0.03	8.31±0.28	3.5±0.3
I382A^7.36^	11.09±0.03	2.59±0.04	9.10±0.05	8.46±0.33	5.0±0.4
K383A^7.37 *^	9.67±0.12 (51)^*†^	0.79±0.18^*†^	8.92±0.03	8.17±0.25	27.0±2.8
L384A^7.38^	10.81±0.05 (4)^*†^	2.23±0.08	8.66±0.01	8.42±0.28	16.9±0.5
F385A^7.39^	11.42±0.09	2.54±0.11	8.94±0.02	8.32±0.32	18.5±0.9
T386A^7.40^	11.17±0.14	2.99±0.17	8.91±0.03	8.36±0 06	3.9±0.28
E387A^7.41 *^	11.03±0.04	1.45±0.05^*†^	9.91±0.02	8.08±0.25	9.3±0.2

### ECL2/TM6

**Figure 3:**
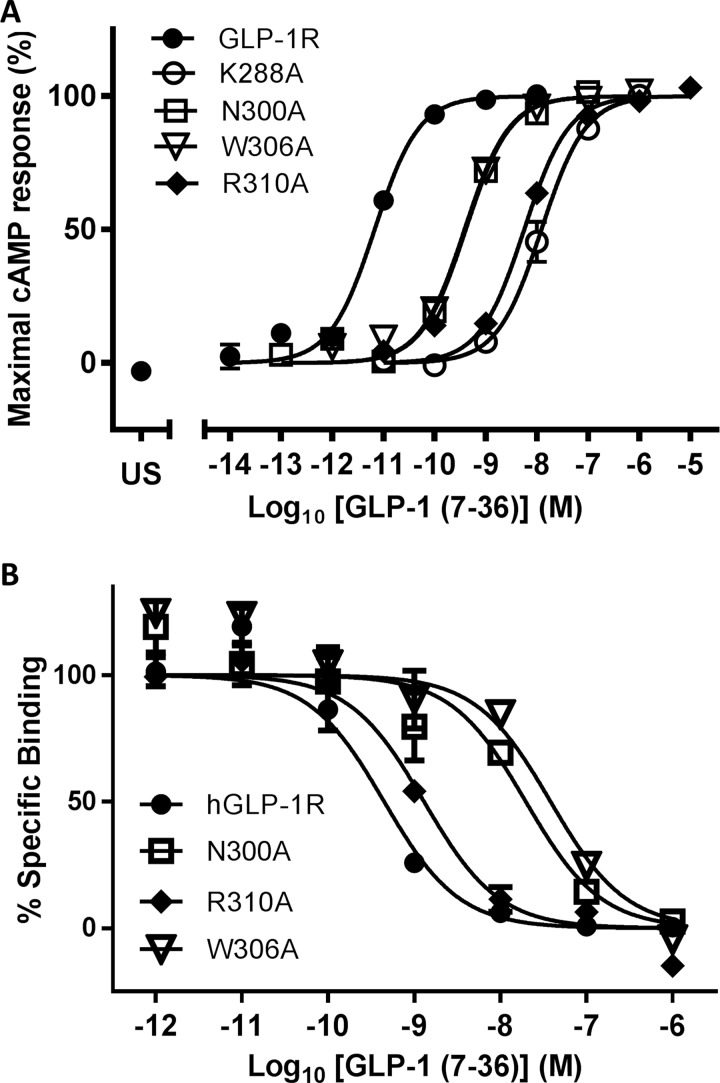
Pharmacological analysis of four mutant receptors compared with wild-type GLP-1R: Lys-288–Ala^4.64^, Asn-300–Ala^ECL2^, Trp-306–Ala^5.36^ or Arg-310–Ala^5.40^ (**A**) Concentration–response curves for GLP-1-induced cAMP accumulation in Flp-In HEK293 cells. US, unstimulated (no GLP-1 added). (**B**) Radioligand competition binding curves, using 75 pM ^125^I-EX4(9–39) and unlabelled GLP-1 as competitor, for membranes derived from Flp-In HEK293 cells expressing either wild-type GLP-1R or one of four single site mutant receptors. Replicates are in triplicate with errors representing S.E.M.

An initial double-Ala scan of the region of hGLP-1R spanning Ile-286^4.62^ to Pro-312^5.42^ (TM4/ECL2/TM5 but excluding the conserved Cys-296^ECL2^; Figure 1B) was carried out using QuikChange mutagenesis. Stable Flp-In HEK293 cell lines were generated expressing each double mutant receptor and LANCE cAMP assays were carried out in order estimate the potency of GLP-1. Membranes derived from these stable cell lines were used in radioligand competition assays to estimate the percentage specific binding of 75 pM ^125^I-GLP-1, using >10,000-fold unlabelled GLP-1 as the competitor (Table S2). Six sites were highlighted by this low resolution pharmacological screen as being potentially interesting, four of which displayed reduced specific binding and a reduced potency of more than 250-fold–hence these sites were analysed in more detail by generating a further eight single Ala mutants. Flp-In HEK293 cell lines expressing each of these eight single mutant receptors were analysed using LANCE cAMP assays and full competition binding analysis with ^125^I-EX4(9–39) as the tracer and both GLP-1 and EX4(9–39) as competitors (Table 1). Although all eight mutant receptors bound EX4(9–39) with the same affinity, five were identified as having GLP-1 affinity and/or potency significantly different from the wild-type receptor (Figure 3): Lys-288^4.64^–Ala displayed a 5248-fold reduction in potency but with only a 23-fold reduction in affinity; Asn-300^ECL2^–Ala displayed a 95-fold reduced GLP-1 potency and 36-fold reduction in GLP-1 affinity; Trp-306^5.36^–Ala displayed a 191-fold reduced GLP-1 potency and 110-fold reduction in GLP-1 affinity and Arg-310^5.40^–Ala displayed a 1175-fold reduction in potency, despite maintaining GLP-1 affinity close to that of wild-type GLP-1R. As might be expected with such large reduction in potency relative to affinity, the operational model predicted that GLP-1 had reduced efficacy at Lys-288^4.64^–Ala and Arg-310^5.40^–Ala (Δlog *τ*_c_=1.39 and 0.75 respectively). Arg-299^ECL2^–Ala also displayed significantly reduced efficacy (Δlog *τ*_c_=0.6). However, log *τ*_c_ values for Asn-300^ECL2^–Ala and Trp-306^5.36^–Ala were not reduced, suggesting that the reduced potency of GLP-1 at these mutants could largely be accounted for by reduced GLP-1 affinity (Figure 3).

### Molecular modelling

The 7TM domain of the GLP-1R closely resembled that of the 4L6R crystal structure and maintained the interactions between the residues that form the base of the cavity through two neighbouring polar clusters that link TM1, TM2, TM6 and TM7. Tyr-152^1.47^, Arg-190^2.60^ and Thr-391^7.45^ form one cluster, whereas His-363^6.52^, Glu-364^6.53^, Glu-387^7.41^, Thr-391^7.45^ and Gln-394^7.49^ form the second. The two networks of interacting side chains were seen to be linked through Thr-391^7.45^ and could potentially also be linked through an interaction between Arg-190^2.60^ and Glu-364^6.53^ via a water molecule, particularly in the ligand-free model. In the docked model, these clusters of polar residues at the base of the binding cavity interact directly with the N-terminal region of GLP-1.

**Figure 4:**
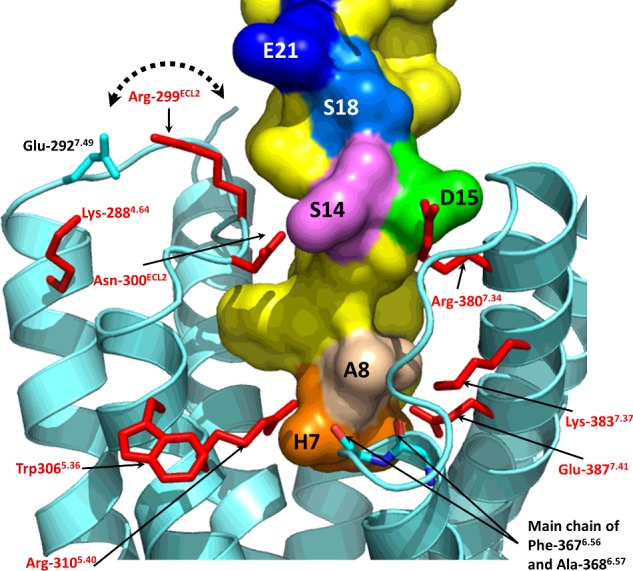
A side view of the GLP-1-docked GLP-1R model from between TM5 and TM6 GLP-1R is shown in cartoon form in cyan, with the side chains of the eight residues highlighted by the mutagenesis (Table 1) shown as red sticks. The ligand is shown with its surface in yellow, with six residues highlighted by colour and single residue codes.

The location and environment of the residues of interest highlighted by the mutagenesis described above are summarized as follows (Figure 4). Both Lys-288^4.64^ and Trp-306^5.36^ do not directly form part of the agonist-binding pocket: Lys-288^4.64^ is shielded from the cavity by ECL2, with which it forms several interactions; Trp-306^5.36^ is on the lipid-facing side of TM5, close to the extracellular end of the helix. The orientation of Arg-299^ECL2^ is ambiguous, with its extended side chain pointing away from the helical bundle where it could be modelled to interact with either Glu-292^7.49^ or Glu-21* and Ser-18*. However, the remaining residues are in closer proximity to the ligand cavity. Asn-300^ECL2^ is able to interact with the side chain of Ser-14*, whereas Arg-310^5.40^ interacts closely with His-7* and is also in close proximity to the free main chain carboxy of Ala-368^6.57^ and the end of TM6. The neighbouring main chain carboxy of Phe-367^6.56^ is also free, and can interact with the side chain of Lys-383^7.37^, which is in the vicinity of Ala-8*. Arg-380^7.34^ at the start of TM7 can interact directly with Asp-15*, which itself hydrogen bonds to Thr-13*. Glu-387^7.41^ interacts with both the main chain carboxy of Ala-8* and the imidazole ring of His-7* (Figure 4).

**Figure 5:**
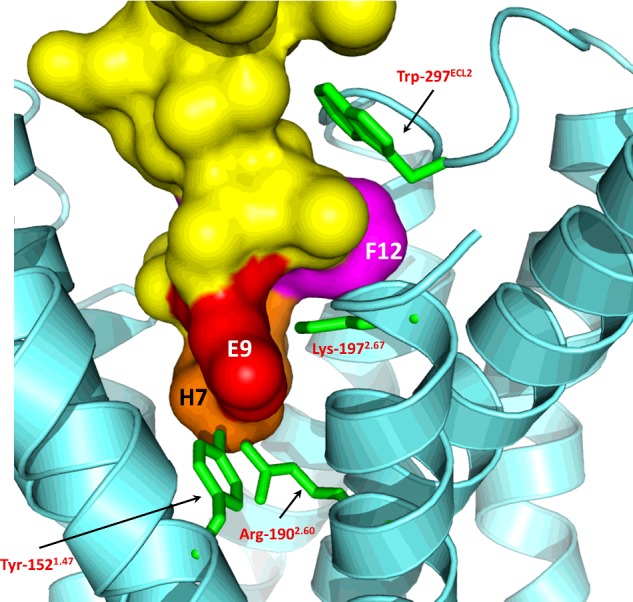
A side view of the GLP-1-docked GLP-1R model from between TM1 and TM2 GLP-1R is shown in cartoon form in cyan, with the side chains of four residues highlighted by mutagenesis in the literature (Table S1) shown as green sticks. The ligand is shown with its surface in yellow, with three residues highlighted by colour and single residue codes.

Other interaction points observed in the model between the peptide and the 7TM domain are as follows (Figure 5). In addition to Arg-299^ECL2^ and Asn-300 ^ECL2^ on ECL2, Trp-297^ECL2^ can interact directly with the ligand and is surrounded by Thr-11*, Val-16* and Leu-20*. Phe-12* sits in a buried pocket underneath ECL2, where it is surrounded by Phe-230^3.33^, Gln-234^3.37^, Thr-298^ECL2^ and Tyr-305^5.35^. The 3^rd^ position of the ligand, Glu-9*, interacts with Trp-152^1.47^, Arg-190^2.60^ and Lys-197^2.67^, with the closest atom–atom distances (excluding hydrogens) all being less than 3 Å (1 Å=0.1 nm). Other residues that form the region of the binding cavity in proximity to Glu-9* are Val-194^2.64^, Met-233^3.36^, Leu-388^7.42^ and Thr-391^7.45^. The N-terminal H-7* of GLP-1 is in a region of the cavity made up of Arg-190^2.60^, Val-237^3.40^, Tyr-305^5.35^, Ile-309^5.39^, Arg-310^5.40^, Glu-364^6.53^, Phe-367^6.56^, Ala-368^6.58^, Glu-387^7.41^ and Thr-391^7.45^. Meanwhile, although important for glucagon recognition in the glucagon receptor [16,24], ECL1 does not play a major role in GLP-1 binding to GLP-1R [25] and was not modelled due to its absence of a template. However, the missing ECL1 loop can be predicted to interact with Gly-10*, Thr-11*, Val 16* and Tyr-19*, as these residues are surface exposed in the vicinity that the missing loop region is likely to occupy.

**Figure 6:**
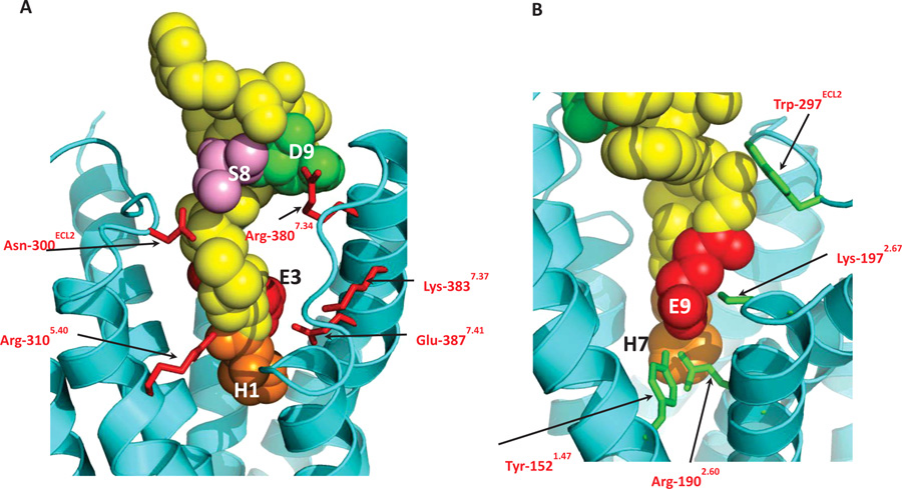
Views of the GLP-1R model docked with “model 11” from pdb code 2N0I (**A**) View of the GLP-1R model from between TM5 and TM6 for comparison with Figure 4. (**B**) View of the GLP-1R model from between TM1 and TM2 for comparison with Figure 5. The ligand is the cyclic constrained synthetic 11-residue analogue of GLP-1 based on model 11 of pdb code 2N0I, and is shown as space-fill in yellow, but with four conserved residues highlighted by colour and single residue codes.

Each of the models from the ensemble in the NMR structure file of pdb code 2N0I, a cyclic constrained synthetic 11-residue analogue of GLP-1 with high potency, was structurally aligned with the GLP-1 ligand in the model. Although it did not share the same backbone conformation as GLP-1, model 11 from the ensemble was seen to align such that four conserved resides (the His-7*, Glu-9*, Ser-14* and Asp-15* equivalents in GLP-1) could be over-laid with the structure of GLP-1 in 3 dimensional space. Model 11 of 2N0I was manually docked into the binding site of the GLP-1R model and found to comfortably occupy the binding cavity without any serious steric issues, while forming very similar interactions with His-7*, Glu-9*, Ser-14* and Asp-15* as seen above with GLP-1 (Figure 6).

## DISCUSSION

The critical region of the peptide ligand responsible for GLP-1R activation has been identified as the extreme N-terminus, truncation of which results in a significant reduction in GLP-1 potency [17]. This region of the peptide agonist interacts with the 7TM domain of GLP-1R and hence it is expected that the ligand–receptor interactions critical for agonist-induced receptor activation will reside in the 7TM region. On the other hand, the N-terminally truncated antagonist EX4(9–39) interacts predominantly with the NTD, hence mutations in the core domain would be expected to have little effect upon its affinity [17]. In the work described here, we explored the interaction between GLP-1’s N-terminal region and the 7TM domain of the receptor by using molecular modelling and Ala-scanning mutagenesis. Despite being the best option available, an inherent problem in using the 7TM domain of the glucagon receptor (4L6R) as a template for an agonist-bound GPCR model is that it probably represents the inactive conformation and hence there must be some conformational movement envisaged when examining the details of the agonist-binding site.

The ligand-docked full-length GLP-1R model differed significantly from that of Siu et al*.* [16] (co-ordinates supplied by Dr Chris de Graaf) both in terms of the relative orientations of the NTD with the 7TM domain, and also in the conformation and placement of the N-terminal region of the agonist within the 7TM helical bundle (Supplementary Figure S3). The principle difference in the relative positions of the ligand and 7TM domain are that the start of the helix (Ser-14*, Asp-15*) in our model inserts into the 7TM cavity between TM7 (Arg-380^7.34^) and ECL2 (Asn-300^ECL2^), whereas the equivalent region in the model of Siu et al*.* inserts between TM7 and the stalk region of TM1. Consequently, the ligand in our model does not interact directly with the stalk region but instead is able to interact more closely with residues in TM5 and TM6, the converse being the case with the model of Siu et al. Furthermore, the conformation of the N-terminal region of the peptide in our model closely matches that of the NMR structure of PACAP21 (pdb code 1GEA), whereas that of Siu et al*.* has a more extended conformation. A consequence of this is that, although the N-terminal His residues in both models are at a similar depth within the 7TM bundle (albeit that it is shifted sideways and so closer to TM5 and TM6 in our model), other residues in the Siu et al. model, such as the equivalent residues to Glu-9* and Phe-12* of GLP-1, are positioned much closer to the extracellular side of the membrane. Therefore, although the generic architecture of the models appears similar at first glance, the molecular details of the binding site are significantly different.

The Ala-scan of ECL3 and the extracellular region of TM7 revealed three residues in TM7 which were involved in agonist recognition–Arg-380^7.34^, Lys-383^7.37^ and Glu-387^7.41^. Arg-380^7.34^–Ala displayed significantly reduced affinity (129-fold) but had wild-type-like efficacy, suggesting that the observed 243-fold reduction in potency was largely due to the affinity loss. This side chain is positioned close to Asp-15* (Figure 6B), an important residue for GLP-1’s affinity and potency [26], which would account for the properties of the mutant receptor. Moon et al. [27] have recently published a study in which Arg-380^7.34^ was replaced by Asp, resulting in almost a 2000-fold reduction in potency. [Arg^9^]-GLP-1, which had almost 100-fold lower potency at wild-type GLP-1R, was shown to have 120-fold improved potency at the Arg-380^7.34^–Asp mutant, a reciprocal rescue which strongly implicates the two residues in an interaction. Furthermore, Moon et al. showed that [Arg^4^]-GLP-1 could also rescue the Arg-380^7.34^–Asp mutant, with a decreased potency of 400-fold at wild type but a 9-fold improved potency at the mutant receptor. In our model, Gly-10* of GLP-1 interacts with Leu-384^7.38^, just one helical turn below Arg-380^7.34^ and therefore an Arg at ligand position 4* could easily reach the Asp substitution at position 380^7.34^, validating the choice of GLP-1 conformation based on PACAP21/1GEA (Figure 6A). Lys-383^7.37^ lies one helical turn below Arg-380^7.34^ and lines the binding cavity. Its substitution by Ala resulted in a significant reduction in GLP-1 efficacy at the mutant receptor, as well as a 50-fold reduction in affinity. In the model, Lys-383^7.37^ is in the vicinity of Ala-8* but too distant (>5 Å) to interact directly with the ligand. This leaves the possibility of there being an intermediate water molecule or, perhaps the model based on the inactive structure of the glucagon receptor template masks a closer interaction between Lys-383^7.37^ and the ligand in the active state. Alternatively, Lys-383^7.37^ could H-bond to the free main chain carboxy of Phe-367 at the end of TM6 which, as discussed below, may form part of a gateway for the entry of the N-terminal region of the peptide into the 7TM bundle. The third residue of interest identified in TM7 was Glu-387^7.41^, which displayed significantly reduced efficacy compared with wild-type GLP-1R which correlates with its location in the model where it interacts directly with the N-terminal His-7* of GLP-1 and also forms part of the polar network at the base of the binding cavity.

In previous studies based on the rat GLP-1R, the importance of both Lys-288^4.64^ and ECL2 for agonist recognition was demonstrated [17,28,29]. A detailed study by Koole et al*.* [30] using human GLP-1R determined the role of specific residues in peptide binding, highlighting some potential differences with the observations in the rat receptor (e.g. Lys-288^4.64^–Ala and Trp-306^5.36^–Ala). In order to examine these differences more closely, we repeated the double Ala scan of ECL2 in human GLP-1R and followed up the most interesting observations with single residue substitutions coupled with a more detailed pharmacological characterization. Four residues in the region encompassing ECL2 and the extracellular ends of TM4 and TM5 were identified as playing a potential role in GLP-1 binding and/or GLP-1-induced receptor activation. In contrast with the observation of Koole et al. [30], Trp-306^5.36^–Ala was expressed as a functional receptor in our system, albeit with 190-fold lower GLP-1 potency. However, the operational model demonstrated that this potency reduction was not accompanied by a significant reduction in efficacy and could hence be explained in large part by its 109-fold reduced affinity for GLP-1. The model suggested this residue to be on the lipid-facing side of TM5 (Figure 4) and close to the phospholipid head-group region (in a similar manner to Trp-284^4.60^, which is also highly sensitive to mutation [33]) where it may play a role in forming a stabilizing tryptophan–lipid cation–*π* interaction [31]. Replacement of such Trp residues with Ala may therefore have an effect on the stability of the receptor, in this case causing an indirect reduction in affinity in Flp-In HEK293 cells but a complete lack of expression in the different membrane environment of Flp-In CHO cells. A second tryptophan residue in ECL2, Trp-297^ECL2^, was identified as being important for affinity and efficacy [30] and in this case the model showed that it interacts directly with both the helical and N-terminal sections of the ligand via Thr-11*, Val-16* and Leu-20* (Figure 5).

Arg-299^ECL2^–Ala displayed reduced efficacy which could be attributed to the removal of a salt bridge interaction with Glu-292^ECL2^, a residue which has also been shown to play a role in GLP-1 efficacy [30]. This putative salt bridge may play an important structural role, the disruption of which could result in a disturbance of the agonist-binding site formed by other residues in ECL2. However, a second possibility is that Arg-299^ECL2^ interacts with the helical region of the ligand (Figure 4). Runge et al*.* [32] showed that the reduced affinity and potency of Ser-12–glucagon (Lys-12* in native glucagon and the equivalent of Ser-18* in GLP-1; Figure 1) at the glucagon receptor could be rescued by the replacement of a region of the sequence (ECL2 and residues in the neighbouring helical regions) with the equivalent from GLP-1R. The model suggests that Arg-299^ECL2^ is the only divergent residue in this region that can contact the ligand in the vicinity of Ser-18*, albeit slightly out of range. If this is the residue responsible for the observation of Runge et al*.*, it suggests a complimentary pairing in GLP-1R between Arg-299 and Ser-18* which is reversed in the glucagon receptor (Ser-297 to Lys-12*).

Another residue which appears to be important for the structural integrity of ECL2 is Lys-288^4.64^, which is not part of the binding cavity but instead appears to have the potential to interact with Glu-292^ECL2^ (Figure 4) and potentially other residues in ECL2 (Thr-298 ^ECL2^, Ser-301 ^ECL2^ and Asn-304 ^ECL2^). We observed a substantial reduction in GLP-1 potency at Lys-288^4.64^–Ala (5289-fold), related to lowered GLP-1 efficacy, which was accompanied by only a 23-fold decrease in GLP-1 affinity. The model suggested that Lys-288^4.64^ may play a critical role in stabilizing ECL2 and hence maintain the correct binding pocket for the binding of the N-terminal region of peptide agonists. Koole at al. [30] observed a 125-fold reduction in affinity and undetectable cAMP activity for the Lys-288^4.64^–Ala mutation in GLP-1R. Although log *τ*_c_ could therefore not be determined in this case, their data also appear to support the reduced efficacy observed at this mutant receptor. However, the Lys-288^4.64^–Ala mutation in rat GLP-1R resulted in a much less substantial loss in potency (250-fold), with a reduction in affinity of 126-fold that was associated only with peptide agonists but not their N-terminally truncated analogues [28].

Although the Asn-300^ECL2^–Ala mutation resulted in 36-fold reduced affinity and 95-fold reduced potency, the operational model indicated no significant reduction in efficacy, while Koole at al. [30] identified this site as being important in both affinity and efficacy. The model locates Asn-300^ECL2^ as being in close proximity to Ser-14* of GLP-1 where it may form a H-bond (Figure 4). A more substantial reduction in potency was observed for Arg-310^5.40^–Ala (over 1000-fold) with a significant reduction in efficacy but accompanied by no significant decrease in GLP-1 affinity. These Arg-310^5.40^–Ala data agree with the study of Coopman et al*.* [33]*,* who demonstrated that the Arg-310^5.40^–Ala mutation resulted in a 1259-fold reduction in potency with only a 10-fold reduction in affinity. Our model places Arg-310^5.40^ in the binding cavity where it can interact directly with the main chain of His-7* (Figure 4). Truncation of the first two residues of GLP-1, to yield GLP-1(9–36)amide, resulted in reduced affinity and a substantial loss of efficacy [17], and hence the residues in the receptor interacting with the extreme N-terminus of the agonist would be expected to be critical for receptor activation. Removal of His-7* and Ala-8* would prevent the peptide interacting with not only Arg-310^5.40^, but also Glu-364^6.53^ and Glu-387^7.41^ which form part of the polar network at the base of the binding cavity. Confidence in the model is further gained since the equivalent residue to Glu-387^7.41^ in the related glucagon receptor (Asp-385^7.41^) has been functionally linked with Ser-2* of glucagon (equivalent of Ala-8* in GLP-1) in a study by Runge et al. [32] in which the mutation Asp-385^7.41^–Glu was shown to improve the affinity and potency of Ala-2–glucagon, despite reducing the affinity and potency of glucagon itself.

In combination with the residues mutated in this and the related studies discussed above, the GLP-1R model also enables the rationalization of other data from the literature in order to explain the agonist-binding site and a basic pharmacophore for GLP-1R peptide agonists. A key residue in the peptide agonist is Glu-9* which is close to the imidazole ring of His-7* in the β-coiled PACAP21-based conformation of GLP-1 (Figure 5). The equivalent residue in the related peptides secretin, VIP and glucagon have been shown to be in close proximity to the GLP-1R equivalents Arg-190^2.60^ and Lys-197^2.67^ [34–38] and this is in complete agreement with the model which has Glu-9* interacting directly with both these basic side chains (Figure 5A). Furthermore, Runge et al*.* [32] showed that the reduced affinity and potency of Glu-3–glucagon (native glucagon has Gln-3*; Figure 1) could be rescued by replacing the sequence in the upper half of the glucagon receptor with GLP-1R, whereas mutagenesis data for GLP-1R have shown that the mutation of both Arg-190^2.60^ and Lys-197^2.67^ to Ala [30,33,39] resulted in reduced affinity of GLP-1. However, the Arg-190^2.60^–Ala substitution did not reduce oxyntomodulin affinity or efficacy, and since oxyntomodulin has Gln at the 3rd position rather than Glu (Figure 1A), this adds further evidence for position 3 of GLP-1 (and its related peptides) being in close proximity to Arg-190^2.60^ and Lys-197^2.67^ (or the corresponding residues in the related receptors). In addition, the model suggested that the phenolic hydroxy group of Tyr-152^1.47^ could also interact with Glu-9*, as well as Arg-190^2.60^, and mutation of this residue reduced affinity by more than 30-fold [33]. The substitution of Thr-149^1.44^, one helical turn above Tyr-152^1.47^, to its SNP variant Met, as well as to several other residue types, had severe effects on affinity and GLP-1 efficacy [40–42]. The model shows that Thr-149^1.44^ interacts with Asp-198^2.68^ which forms a polar interaction that has also been shown to be important in GLP-1 affinity and potency [43], suggesting the importance of the interaction between TM1 and TM2 in maintaining the structural integrity of the binding site close to the interaction site with Glu-9*. Overall, the model suggests that the N-terminus of GLP-1 binds to several polar residues close to the base of the binding pocket where it is likely to disrupt the polar networks and so trigger conformational change and receptor activation.

An interesting and necessary issue to consider in defining the peptide-binding site of GLP-1R is that of GLP-1(1–36)amide, the physiological N-terminally extended precursor to GLP-1, which is a full agonist at GLP-1R with 50-fold lower potency [30]. Given that His-7* of GLP-1 is buried within the 7TM bundle, how can the additional six N-terminal residues be accommodated? Analysis of the GLP-1-bound GLP-1R model shows that part of the agonist-occupied binding cavity remains empty and that this ‘residual pocket’ is accessible via an opening situated between the top of TM5 (Asn-300^ECL2^, Asn-302^ECL2^, Trp-306^5.36^) and the TM6/ECL3 interface (Phe-269^4.45^, Asp-372^4.48^). The residual pocket is lined by Asn-300^ECL2^, Asn-302^ECL2^, Tyr-305^5.35^, Trp-306^5.36^, Arg-310^5.40^, Phe-367^6.56^, Ala-368^6.57^, Phe-369^6.58^, Met 371^ECL2^, Asp-372^ECL2^, Arg-380^7.34^ and Lys-383^7.37^, with the base being formed by two free main chain carboxy groups at the C-terminal end of TM6. Koole et al. [30] have shown that the mutation of several of residues in ECL2 resulted in selective effects on GLP-1(1–36)amide–in particular, Tyr-305^5.35^–Ala has no significant effect on GLP-1 efficacy but resulted in a substantial reduction in the efficacy of GLP-1(1–36)amide (Δlog *τ*_c_=1.08). We propose that the N-terminal extension of GLP-1(1–36)amide is accommodated in the binding site through the doubling back of the N-terminal region of the peptide chain, which changes direction at His-7* at the base of the cavity, and then travels back up through the residual pocket and out through the opening where the N-terminal residues would be expected to reach the extracellular environment (Supplementary Figure S4).

Yang et al. [44] have recently highlighted the mobility of the NTD relative to the 7TM domain, mediated through the stalk region, and the stabilization of an open conformation by agonist binding. The implication is that the inactive conformation is a closed state and such a model would help to explain the properties of a non-peptidic antagonist T-0632 [45], the binding site of which has been linked with Trp-33^NTD^ on the external solvent-accessible face of the helix of the NTD, where it is difficult to envisage its antagonistic mechanism. However, the consideration of a closed state in which the NTD interacts with the 7TM domain provides the possibility of an antagonist-binding site composed of residues on both domains and a mechanism of antagonism based upon stabilizing the closed state and thereby impeding agonist action, rather than directly blocking the agonist-binding site (Supplementary Figure S5). Furthermore, the mobility of the ligand-bound NTD may enable the N-terminal region of the peptide to come into transient contact with sites on the 7TM domain outside the binding site, which may explain some of the benzoylphenaline cross-linking data that predicts some peptide–receptor interactions that, taken together, provide distance restraints which are difficult to reconcile both with each other and with the current model of the GLP-1R-binding site [46–48] (Supplementary Figure S5).

Following the first stage in the two domain model for Family B GPCR agonist recognition, the ligand is bound to the NTD. To complete the second stage, the N-terminus needs to gain access to the cavity, and, given the constraints posed by it being bound to the NTD, one way this could be achieved is via a ‘pendulum-like’ motion in which the NTD–ligand complex rotates via a pivot point in the stalk, and moves the N-terminal region of the ligand through an opening between TM5 and TM6/ECL3 and into the main cavity (Supplementary Figure S5). An opening between TM5 and TM6 can be seen both in the glucagon receptor crystal structure and also in the CRF_1_ receptor, the other known structure of the 7TM domain of a Family B GPCR, suggesting that this feature may be common across the class (Supplementary Figures S6A and S6B). The transition of the N-terminal region of the peptide through this gap would explain the disulfide-trapping data obtained from the related PTH_1_ receptor in which four residues at the TM5/TM6 interface were each individually mutated to Cys and then disulfide cross-linked to an analogue of parathyroid hormone (PTH) with Cys at position 1 [49]. The equivalent positions in the 4L6R crystal structure reveal that one of these residues (Leu-368^5.44^–Cys) is on the lipid-facing site of TM5 and hence inaccessible to the N-terminal region of the ligand from the main cavity in the 7TM domain (Supplementary Figure S7). However, passage of the N-terminal region through the opening between TM5 and TM6/ECL3 would explain how the Cys at position 1 of the ligand was able to come into contact with the lipid-facing cysteine side chain at residue position 5.44.

In conclusion, we have constructed a molecular model of GLP-1R which combines the known information from several crystallographic and NMR studies, and we have used a wealth of pharmacological data generated from site-directed mutagenesis of GLP-1R to inform and explain the docking of the N-terminus of GLP-1 into the cavity of the 7TM domain of the receptor. Further examination of the model enabled an informed prediction of how residues 1*–6* of the N-terminally extended ligand GLP-1(1–36) could be accommodated in the binding site, as well as identifying the likely mechanism for entry of the N-terminal region of the ligand into the 7TM domain cavity. Identification of key interaction points between the ligand and receptor were used to predict a basic pharmacophore and to dock an 11-residue analogue of GLP-1 into the receptor-binding site, despite it having a different main chain conformation to GLP-1 itself. The model (model co-ordinates available from corresponding author upon request) provides a valuable tool for the prediction and rationalization of further experiments.

## Abbreviations

ECL: extracellular loop
GLP-1: glucagon-like peptide-1 (7–36)amide
GLP-1R: glucagon-like peptide-1 receptor
GPCR: G protein-coupled receptor
HEK: human embryonic kidney
MBS: membrane-binding solution
NTD: N-terminal domain
PACAP: pituitary adenylate cyclase-activating protein
PTH: parathyroid hormone
TM: transmembrane
